# Amplitude setting and dopamine response of finger tapping and gait are related in Parkinson’s disease

**DOI:** 10.1038/s41598-022-07994-8

**Published:** 2022-03-09

**Authors:** Hafsa Bareen Syeda, Aliyah Glover, Lakshmi Pillai, Aaron S. Kemp, Horace Spencer, Mitesh Lotia, Linda J. Larson-Prior, Tuhin Virmani

**Affiliations:** 1 Department of Neurology, University of Arkansas for Medical Sciences, 4301 W. Markham St., #500, Little Rock, AR 72205-7199 USA; 2 Department of Psychiatry, University of Arkansas for Medical Sciences, Little Rock, AR USA; 3 Department of Biostatistics, University of Arkansas for Medical Sciences, Little Rock, AR 72205 USA; 4 Department of Neurobiology and Developmental Sciences, University of Arkansas for Medical Sciences, Little Rock, AR USA; 5 Center for Translational Neuroscience, University of Arkansas for Medical Sciences, Little Rock, AR 72205 USA

**Keywords:** Parkinson's disease, Movement disorders

## Abstract

Movement amplitude setting is affected early in Parkinson’s disease (PD), clinically manifesting as bradykinesia. Our objective was to determine if amplitude setting of upper limb bimanual movements and bipedal gait are similarly modulated in PD. 27 PD and 24 control participants were enrolled. Participants performed a bimanual anti-phase finger tapping task wearing gloves with joint angular sensors, and an instrumented gait assessment. Participants performed normal and fast paced assessments to vary motor load. PD participants were evaluated OFF (PD-OFF) and ON (PD-ON) levodopa. PD-OFF participants had smaller tap amplitude, and greater tap amplitude variability than controls in the more affected hands (all *p* < 0.05). Tap amplitude and stride length (*p* = 0.030) were correlated in PD-OFF. Tap amplitude was also correlated with motor UPDRS (*p* < 0.005) and bradykinesia motor (*p* < 0.05) and ADL (*p* < 0.005) UPDRS subscores. The relative amount of improvement in tap amplitude and stride length with levodopa was correlated. In PD, upper limb and gait amplitude setting are similarly scaled with motor demand and dopamine supplementation. This suggests these automated motor functions are subserved by common functional networks.

## Introduction

Bradykinesia is defined as a slowing of movement in addition to a tapering amplitude with repetitive movements^1^. By contrast hypokinesia refers to slowing of movement alone^2^. Limb bradykinesia, based on the UK brain bank^3^ and MDS criteria^4^, is required for a clinical diagnosis of Parkinson’s disease (PD). Upper limb bradykinesia can lead to significant functional decline in tasks requiring bimanual coordination such as cutting meat, buttoning clothes and shampooing one’s hair^2^. While these tasks may not have the same rhythmicity of movement that gait does, they are still learned complex patterned automatic movements that do not require significant thought during their performance.

Studies to address deficits in upper limb movements have been performed using in-phase (limbs moving together in the same direction) and out-phase (limbs moving in opposite directions) movements^5,6^, using proximal arm displacements towards and away from the body^5^, pronation/supination, wrist flexion-extension^7^, and circular drawing movements^6^. More complex tasks such as moving one’s hand to the mouth have also been explored^8^. Mechanical sliders^5^, tablets^9^, MIDI keyboards^10^, and 3D motion capture^11^ have been utilized as measurement tools. Importantly, upper limb bimanual coordination deficits have been shown to occur early in the PD disease course^6^ and exhibit more difficulty in anti-phase movements^5,12^.

Parkinson’s disease affects motor function in the limbs and gait, but studies comparing unconstrained upper and lower limb function in the same participants are limited. This is necessary to determine whether amplitude setting in both limbs is similarly affected by disease and similarly modulated by dopamine. Using a task instructing participants to move their finger between two dots a fixed distance apart, Williams et al.^13^ found that upper limb and gait phase coordination indices were correlated, where upper limb speed was constrained by a metronome timed to individual gait cadence. In early PD participants, Delval and colleagues^14^ constrained finger and foot tapping movements to set metronome frequencies, but not gait speed, and found limb freezing and festination episodes before gait freezing in some participants, suggesting a break down in repetitive movements before more complex movements such as gait. Both these studies were performed OFF-levodopa, and since dopamine supplementation improves some but not all motor features of PD^15^, determining whether amplitude setting responds similarly in both limbs to dopamine is important. Barbe and colleagues^16^ performed OFF- and ON-state measurements in finger tapping and gait but did not directly compare their measures in these four conditions. They found that smaller step length and variability in upper limb movements was related to the occurrence and duration of gait and upper limb freezing respectively. The direct kinematic relationships between finger tapping, foot tapping, and gait were not explored in these studies.

No studies have been conducted to our knowledge in people with Parkinson’s disease to determine whether the amplitude setting of upper limb movements and gait show similar deficits. As upper body parkinsonism is a more common early disease manifestation than gait deficits^2^, evaluation of pathways that are disrupted at the earliest stages of PD using finger tapping paradigms could provide earlier insight into putative targets for pharmaceutical intervention or neuromodulation. An upper limb measure that relates to changes in gait would also allow easier study of pathways subserving these movements using imaging modalities.

We hypothesized that both upper limb bimanual movements and gait movement amplitude would be similarly modulated in PD participants. If this were true then we would expect that finger tap amplitude would be reduced proportionally with gait stride length in PD participants. We would also expect movement amplitude in both paradigms to be similarly modulated by increased motor demand and dopamine supplementation. To test this hypothesis, we had healthy age matched controls and PD participants (in both the levodopa OFF- and ON-state) perform bimanual anti-phase finger tapping wearing data gloves (with a sensor on the metacarpophalangeal joint) and walk on an instrumented gait mat at normal and fast speeds.

## Methods

### Standard protocol approvals, registrations, and participant consents

Participants were recruited from the Movement Disorders Clinic at the University of Arkansas for Medical Sciences (UAMS). The study was approved by the UAMS institutional review board (UAMS IRB# 228861), written informed consent was obtained from all participants, and the study was conducted in accordance with the guidelines of the Declaration of Helsinki.

#### Study population

Participants with PD based on UK brain bank diagnostic criteria^3^, and age-matched controls (controls) between the ages of 45–90 were enrolled. 51 participants were enrolled (24 controls, 27 PD) and analyzed. Exclusion criteria included inability to walk on the Zeno walkway, falls > 1/day, cognitive impairment sufficient to impair capacity for informed consent, diagnosis of a neurologic disorder (other than PD for the PD group), diagnosis of a psychiatric disorder other than those associated with PD, the use of anti-dopaminergic medications in the year prior to enrollment, chronic back, hip or knee pain that was not controlled, severe osteoarthritis, hip or knee replacement surgery or spine surgery in the last 12 months or complicated by persistent pain, and inability to complete questionnaires in English. A complete Unified Parkinson’s Disease Rating Scale (UPDRS), a Hoehn and Yahr staging score (H&Y), the Montreal Cognitive Assessment (MoCA)^17^, and the Hamilton depression (HAM-D)^18^ and anxiety (HAM-A)^19^ rating scales were also performed on all participants. UPDRS Bradykinesia motor (sum of UPDRS items 23–26 and 31) and activities of daily living (ADL) (sum of UPDRS items 9–12) subscores were calculated for PD participants.

### Disease asymmetry calculations

**Figure 1:**
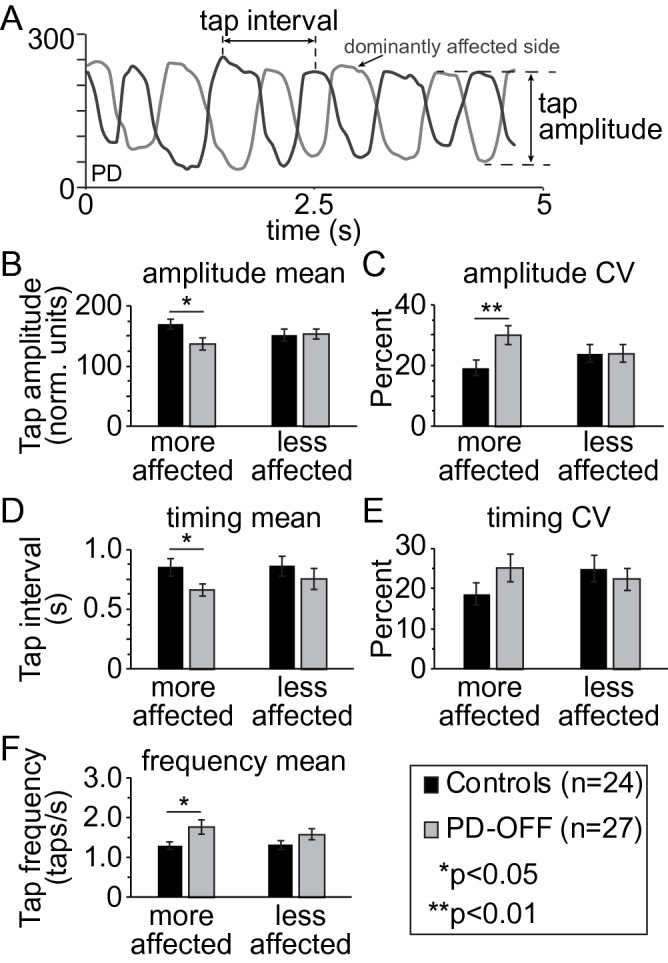
Spatiotemporal parameters of upper limb finger tapping. (**A**) Sample trace showing bimanual finger tapping output using the data glove in a participant with PD in the levodopa OFF state. Definitions of tap interval and tap amplitude are shown. Mean and variability (CV) in finger tap amplitude (**B**, **C**) finger tap interval (**D**, **E**), and tap frequency (**F**) for the more affected (MA) and less affected (LA) hands are shown for controls and PD participants OFF levodopa (PD-OFF). Bar graphs are plotted as the mean ± SEM.

In PD participants, the side more affected (MA) by PD was calculated based on the ratio of the summated right/left scores for items 20–26 of the UPDRS corresponding to tremor, bradykinesia and rigidity, with a score > 1 indicating right-side more affected by PD. All data are reported for the more affected (MA) side, except in Fig. 1, which also shows results from the less affected (LA) side.

### Upper limb dynamics

Participants were placed in a comfortable seated position at a desk with data gloves on (5DT Data Glove, Fifth Dimension Technologies Inc., Orlando, FL), instructed to rest the palms of their hands on the table and alternately tap the right index finger and left index finger on the table; i.e. while the right index finger was moving towards the table, the left index finger would be concurrently lifting off the table (out-of-phase tapping) and vice-versa. This task was chosen to simulate bipedal gait where the right and left leg alternately move forward in a patterned manner, with one leg advancing at a time. As the other leg remains planted for the majority of the gait cycle during which the other leg is moving forward, it is effectively moving backwards in relation to the center of gravity. This task was performed for 20 s at (1) a “comfortable” pace (normal speed) and (2) as fast as possible (fast speed). The normal speed task was performed to mimic normal speed of gait and was not constrained to a metronome as has been done in some prior studies^13,20^, as gait studies are also not constrained to a metronome unless auditory cuing tasks are being tested. Alternating tapping movements were confirmed by visual examination during the task. The fast speed tapping was performed to test the response of participants’ movement to a task requiring increased motor complexity or motor load. The same motor loading task was performed for gait by asking participants to walk at a fast speed.

Data was recorded using 5DT Glove Manager Software, which generates a CSV data file. The glove uses an 8-bit A/D convertor providing 256 intermediate positions between a flat and fisted hand. An intrinsic algorithm in the software adjusts the raw values collected to the maximum and minimum sensor angles, with the hand flat and fisted positions to account for the effect of varying hand sizes^21^. To calculate tap interval and tap amplitude of right and left index finger from these sinusoidal recordings, a protocol using Python and Visual Basic for Applications (VBA) was developed. Finding peaks in a signal depends on distinguishing between actual peaks and noise /baseline changes. Considering the noisy signals, we decomposed the peak detection procedure into two parts: calculating an amplitude threshold for peak selection and peak/trough detection. For amplitude threshold selection, the average of normalized glove readings was found to be too high to optimize the peak detector to distinguish between actual peaks and noise. Upon further iterations, the absolute difference between frames/sets of 10 values and the absolute difference between individual values was calculated and averaged. The difference between the two averages produced the most accurate threshold for peak detection. Using this threshold, peaks and troughs were detected by the local maxima and local minima method^22^ and automated in VBA for all participant files. Python Pandas and Numpy libraries were used for peak/trough finding. The signal was then plotted against time with marked peaks and troughs using Matplolib.

The difference between the value of the trough of the sinusoidal curve to the next peak was used to calculate the amplitude of each finger tap (see Fig. 1A). This value is a normalized output with a range from 0 to 255 units based on the flat and fisted configuration of an individual’s hand and will be referred to in terms of normalized units. The mean and percent coefficient of variability (CV) for finger tap amplitude across each trial was calculated independently for each hand. Two timing variables were also calculated in addition to tap amplitude to compare and contrast any changes in amplitude measures. The tapping frequency was calculated as the number of taps divided by the 20 s trial duration. The mean and CV intertap interval was calculated independently for each hand from the time interval between the peaks on the sinusoidal tapping traces for each finger tap (see Fig. 1A). As phase coupling between hands (and legs for gait) was not the primary interest of this study, the hands were analyzed independently—any phase changes that may have occurred during the tapping task were not considered in the analysis.

### Gait kinematics

Participants were instructed to walk at (1) a “comfortable” pace (normal speed) and (2) as fast as possible (fast speed), 8 lengths of a 20’ × 4’ instrumented gait mat (Zeno Walkway, Protokinetics, Haverton, PA), and data was collected and analyzed using the Protokinetics movement analysis software (PKMAS)^23,24^. The two speeds were chosen to correspond to the normal and fast speed (to assess motor load) performed during finger tapping. The mean and CV for continuous gait stride length, and stride time as well as gait cadence (steps/minute), were extracted using the intrinsic algorithms of PKMAS as in prior published studies^25,26^. These variables were chosen as amplitude, timing and frequency gait variables to correspond to the finger tapping measures defined above.

### Dopaminergic response

All PD participants underwent UPDRS, upper limb dynamics and gait kinematic measurements in the morning in their effective levodopa OFF-state after withholding their Parkinson’s medications overnight as per prior protocols (PD-OFF)^13,14^. PD participants who were taking levodopa (n = 23) as part of their clinical regimen were examined again 60 min after their regular morning dose of levodopa (PD-ON) in order to determine if amplitude setting in the upper limb and gait both responded similarly to levodopa in individual participants. Participants were not excluded from participating if they were not clinically treated with levodopa as the majority of the analysis was performed in the levodopa OFF-state.

### Statistical analysis

Statistical analysis was performed using SPSS 24 (IBM). Our primary interest was comparing mean and variability in amplitude measures in the upper limb (finger tap amplitude) and gait (stride length). Timing and frequency measures were also compared as a contrast to the amplitude measures. Linear regression analysis was used to compare objective spatiotemporal gait and finger tapping measures in the PD group. As the groups were not balanced for gender, we used gender as an independent variable in a model with each of the variables of interest (example finger tapping amplitude) as the dependent variable and calculated the residuals. The gender adjusted residuals from the variables of interest were then compared in a linear regression model as well. In order to determine the strength of the correlation, either Pearson’s or Kendall’s tau correlation coefficients were also calculated. A post-hoc Benjamini–Hochberg adjustment was applied for the multiple comparisons performed in Table 3. A student’s t-test was used to compare the control and PD group differences. Secondary interest was to determine if amplitude setting in upper and lower limb movements were similarly regulated when exposed to a motor load through speeded movements, or dopamine repletion through measurement of levodopa responsiveness (OFF–ON dopaminergic medications). A paired student’s t-test was used to compare results in the OFF-state and ON-state in the subset of 21 PD participants who completed both assessments. Repeated measures analysis of variance (ANOVA) was utilized to calculate the combined group (control/PD) and speed of tapping (normal/fast speed) differences.

## Results

Age was well matched between the PD and control groups (PD 69.3 ± 8.4 years, controls 66.5 ± 8.1 years, *p* = 0.228) but the gender distribution was opposite in the two groups (PD 29.6% female, controls 66.7% female, *p* = 0.008) as most controls were spouses of the PD participants (Table 1). PD participants had a mean baseline MoCA score that was two points lower than controls (PD 26.1 ± 3.4, controls 28.0 ± 1.7, *p* = 0.013) although the mean score in both cases remained in the normal range for the test (≥ 26) (Table 1). Depression and anxiety scores were also higher in the PD group (Table 1). Other features of the PD group are also noted in Table 1.

**Table 1:** Demographics.

	HC (n = 24)	PD (n = 27)	*p* value
Age at enrollment (years)	66.5 ± 8.1	69.3 ± 8.4	0.228
Gender (female/male)	16/8	8/19	**0.008**
Right-handed	100%	81%	0.085
OFF UPDRS Part III (motor) Score	4.1 ± 3.4	24.4 ± 11.2	** < 0.001**
OFF Total UPDRS Score	6.7 ± 4.5	40.1 ± 18.5	** < 0.001**
ON UPDRS Part III (motor) Score		16.6 ± 10.3 (n = 22)	
ON Total UPDRS Score		28.1 ± 17.4 (n = 22)	
Motor UPDRS (OFF–ON)		7.8 ± 6.9 (n = 22)	
MOCA score	28.0 ± 1.7	26.1 ± 3.4	**0.013**
HAM-D	2.9 ± 3.2	6.3 ± 4.7	**0.004**
HAM-A	2.7 ± 3.0	4.7 ± 3.6	**0.018**
Right side more disease affected (MA)	–	56%	
PIGD phenotype at visit	–	41%	
Initial symptom (rest tremor/gait)	–	48%/15%	
Freezing of gait reported at visit	–	37%	
Age at onset (years)	–	60.1 ± 10.3	
Disease duration (years)	–	9.2 ± 5.7	
Hoehn & Yahr score	–	2.1 ± 0.8	
Daily levodopa dose (mg/day)	–	667 ± 368 (n = 23)	
levodopa per dose (mg/dose)	–	194 ± 84 (n = 23)	
Duration on levodopa (years)	–	5.3 ± 3.2 (n = 23)	
On dopamine agonist at visit	–	7%	
On MAO-I at visit	–	33%	
LEDD (l-dopa + agonist + MAO-I)	–	695 ± 393 (n = 24)	

Significant *p* values are in bold.

### Gender and disease asymmetry of finger tapping spatiotemporal parameters

The output of the data gloves provides a sinusoidal trace of finger tapping as shown in Fig. 1A for a PD participant. Tap amplitude (trough to peak, tap amplitude) and intertap interval (peak-to-peak time, illustrated as tap interval) measures are illustrated in Fig. 1A. Additional sample traces of control participants and PD participants both OFF and ON levodopa are shown in Supplementary Fig. 1. As the gender distribution was different between the PD and control groups (Table 1), we explored finger tapping kinematics by gender in each group. No significant differences in any measure of upper limb kinematics was found between genders in either group (Table 2), so measures were collapsed across gender.

**Table 2:** Gender grouped upper limb kinematics.

	Controls			PD		
	Female (n = 16)	Male (n = 8)	*p* value	Female (n = 8)	Male (n = 19)	p-value
Mean tap amplitude (norm. units)	164.49 ± 46.09	182.27 ± 27.33	0.250	132.97 ± 56.41	139.36 ± 51.27	0.787
CV tap amplitude (%)	20.06 ± 12.02	17.48 ± 13.22	0.651	20.06 ± 12.02	17.48 ± 13.22	0.651
Mean tap interval (s)	0.76 ± 0.25	1.05 ± 0.47	0.140	0. 65 ± 0.27	0.67 ± 0.28	0.883
CV tap interval (%)	18.70 ± 12.69	18.70 ± 16.97	0.999	24.39 ± 18.36	25.37 ± 17.91	0.901
tap frequency (taps/s)	1.40 ± 0.50	1.08 ± 0.58	0.211	1.64 ± 0.56	1.83 ± 1.09	0.559

All values reported as mean ± standard deviation.

Baseline upper limb kinematic features between control and PD participants in the OFF levodopa state (PD-OFF) are illustrated in Fig. 1. At a self-defined normal tapping speed, there were significant differences in the more affected (MA) but not less affected (LA) hand in PD-OFF compared to control participants. For amplitude measures in the MA hand, PD-OFF had a smaller mean tap amplitude (Fig. 1B) and greater CV tap amplitude compared to controls (Fig. 1C). For our secondary comparisons, mean tap interval was also shorter (Fig. 1D), and tap frequency faster (Fig. 1F) in PD-OFF compared to controls. PD participants therefore tended to show more tachykinetic (smaller amplitude, faster tapping) finger tapping than controls. As group differences were only observed in the more affected hand during the bimanual tapping task, all subsequent analysis is shown for the more affected hand only.

### Comparison of finger tapping and gait spatiotemporal parameters

**Figure 2:**
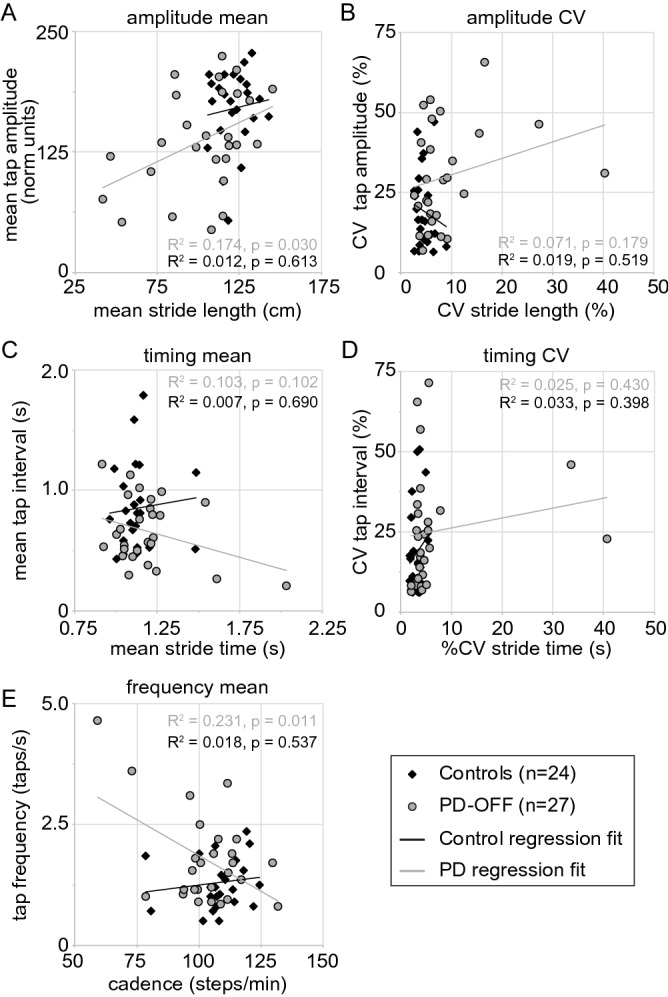
Spatiotemporal parameters of upper limb finger tapping and gait for the more affected limb. Mean and variability (CV) measures for finger tap amplitude vs stride length (**A**, **D**) finger tap interval vs stride time(**B**, **E**), and tap frequency vs cadence (**C**) for self-defined normal “comfortable” paced movements of the dominant limb are shown for controls (black diamonds) and PD participants OFF levodopa (PD-OFF). Each symbol represents a single participant. Best fit lines for PD and control participants are also shown.

To determine the relationship between upper limb and gait kinematics, we examined finger tapping and gait in the more affected hand and leg in PD-OFF participants. Our primary comparison was in the amplitude measures of finger tapping and gait (Fig. 2A,B). Using linear regression analysis mean stride length was able predict mean tap amplitude (F(1, 25) = 5.278, *p* = 0.030, R^2^ = 0.174) and the correlation was positive (Pearson’s = 0.418) (Fig. 2A). Gender adjusted unstandardized residuals for tap amplitude and stride length were also significantly related (F(1, 26) = 5.211, *p* = 0.031, R^2^ = 0.172; Pearson’s = 0.415).

Secondary analysis was performed comparing tap amplitude to other gait measures, UPDRS and MoCA scores (Table 3). In PD-OFF participants tap amplitude was inversely correlated with motor and total UPDRS scores and also with the bradykinesia subscores of the motor (sum of UPDRS items 23–26 and 31) and ADL sections (sum of UPDRS items 9–12), with only bradykinesia motor subscore not significantly correlated after adjustment for multiple comparisons using a Benjamini–Hochberg adjustment.

**Table 3:** Kendall’s tau correlation coefficients comparing disease dominantly affected upper limb kinematics, gait and UPDRS scores.

		Cadence (steps/min)	Mean stride time (s)	Mean stride length (cm)	CV stride time (%)	CV stride length (%)	OFF Motor UPDRS	OFF Total UPDRS	OFF brady motor UPDRS subscore	OFF brady ADL UPDRS subscore	MoCA
**Secondary comparisons**											
Mean tap amplitude (norm. unit)	Control	− 0.091, 0.535	0.066, 0.655		− 0.011, 0.941	0.054, 0.710	0.192, 0.202	0.182, 0.222	–	–	0.085, 0.588
	PD	0.054, 0.692	− 0.063, 0.646		− 0.242, 0.076	− 0.185, 0.175	− 0.375, 0.006*	− 0.396, 0.004*	− **0.360,** **0.011**	− **0.390,** 0.006*	0.135, 0.342
**Exploratory comparisons**											
CV tap amplitude (%)	Control	0.069, 0.637	− 0.044, 0.766	− 0.025, 0.862	− 0.025, 0.862	− 0.105, 0.472	− 0.222, 0.140	− 0.204, 0.171	–	–	− 0.037, 0.817
	PD	0.043, 0.755	− 0.023, 0.868	− 0.151, 0.269	0.225, 0.100	0.179, 0.189	**0.312, 0.023**	**0.287, 0.037**	**0.289,** **0.041**	0.266, 0.059	− 0.051, 0.720
Mean tap interval (s)	Control	− 0.130, 0.372	0.135, 0.358	0.203, 0.165	− 0.007, 0.960	0.152, 0.298	0.045, 0.764	− 0.033, 0.823	–	–	0.073, 0.643
	PD	0.077, 0.574	− 0.074, 0.588	0.202, 0.139	− 0.254, 0.064	− 0.185, 0.175	− 0.198, 0.150	− **0.281, 0.041**	− 0.134, 0.343	− **0.337,** **0.017**	0.111, 0.435
CV tap interval (%)	Control	0.094, 0.519	− 0.062, 0.673	0.138, 0.346	0.043, 0.766	0.072, 0.620	− 0.203, 0.177	− 0.211, 0.156	–	–	0.138, 0.381
	PD	− 0.066, 0.632	0.074, 0.588	− 0.259, 0.058	0.208, 0.128	0.128, 0.348	0.226, 0.099	0.195, 0.156	0.265, 0.061	0.089, 0.529	− 0.117, 0.410
Tap frequency (taps/s)	Control	0.169, 0.253	− 0.173, 0.242	− 0.147, 0.320	− 0.029, 0.842	− 0.147, 0.320	− 0.046, 0.764	− 0.007, 0.960	–	–	− 0.049, 0.757
	PD	− 0.081, 0.559	0.078, 0.573	− 0.219, 0.112	0.242, 0.079	0.179, 0.195	0.229, 0.099	**0.294, 0.035**	0.175, 0.220	**0.341,** **0.016**	− 0.140, 0.331

Data reported as: Kendall’s tau correlation coefficient, *p* value; significant correlations are in bold.

*correlations that were significant (*p* < 0.05) after application of a Benjamini–Hochberg adjustment.

Exploratory analysis was undertaken to compare the finger tapping and gait measures of timing (Fig. 2C,D) and frequency (Fig. 2E) of movement. In PD-OFF, using linear regression analysis, gait cadence predicted tap frequency [F(1,26) = 7.528, *p* = 0.011, R^2^ = 0.231) and correlation was inverse (Pearson’s = − 0.481) (Fig. 2E)]. Gender adjusted unstandardized residuals for tap frequency and gait cadence were also significantly related (F(1, 26) = 8.254, *p* = 0.008, R^2^ = 0.248; Pearson’s = − 0.498).

### Finger tapping manipulating motor-load

**Figure 3:**
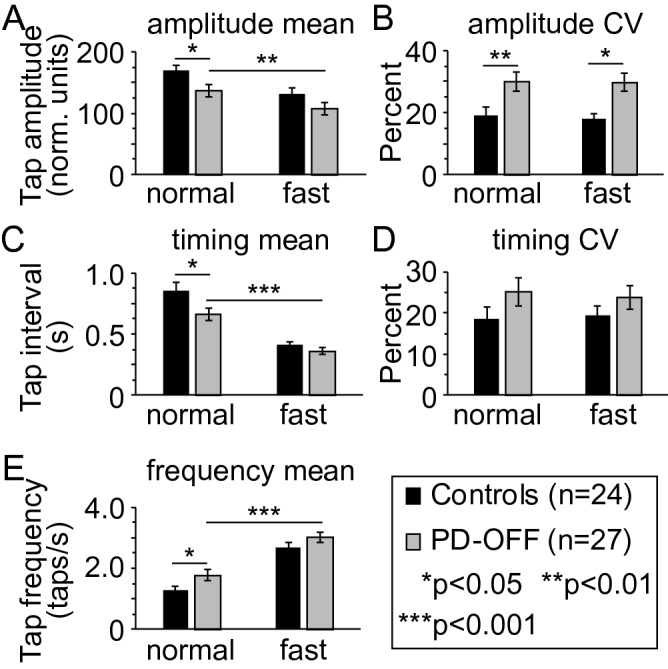
Spatiotemporal parameters of upper limb finger tapping manipulating tap speed in the more affected hand. Mean and variability (CV) in finger tap amplitude (**A**, **B**) finger tap interval (**C**, **D**), and tap frequency (**E**) for self-defined normal (Norm) and fast paced (Fast) finger tapping are shown for controls and PD participants OFF levodopa (PD-OFF). Bar graphs are plotted as the mean ± SEM.

In order to increase motor load, participants were asked to tap at a self-defined fast speed (Fig. 3). Finger tapping was not entrained to a particular metronome frequency, as typically gait assessments are also not set to any particular frequency and we did not want to constrain variability in the responses. In both PD-OFF and controls, there was a smaller mean tap amplitude (Fig. 3A) in the fast compared to normal speed. Using a 2 × 2 multifactorial ANOVA with speed (fast-slow) and group (PD-OFF/controls) as factors there was a between-subject main effect for tap amplitude (*p* = 0.018) but no within-subject effect for speed × group. Tap amplitude CV was higher in PD-OFF compared to control at both speeds (Fig. 3B) but there was not a statistically significant between-subject main effect (*p* = 0.018) or a within-subject effect for speed × group.

On secondary measures, compared to normal speed, PD-OFF had a shorter mean tap interval (Fig. 3C) and faster tap frequency (Fig. 3E) at the faster tap speed. There were between-subject main effects for mean tap interval (*p* = 0.033) and tap speed (*p* = 0.034), but not CV tap interval (*p* = 0.139). There were no within-subject effects for speed × group.

### Levodopa response in finger tapping and gait spatiotemporal parameters

As dopamine replacement helps treat motor symptoms in PD, we wanted to assess the relative effects of levodopa on finger tapping and gait dynamics in the same group of people. If the dopamine response was related in the two assessments, then this would suggest that they may be subserved by the same dopaminergic pathways. Six PD-OFF participants were not included in this analysis due to the following: 4 were not clinically treated with levodopa for symptomatic management, 1 participant forgot to bring levodopa to the visit, and 1 participant’s ON-levodopa finger tapping data was corrupted and could not be used.

**Figure 4:**
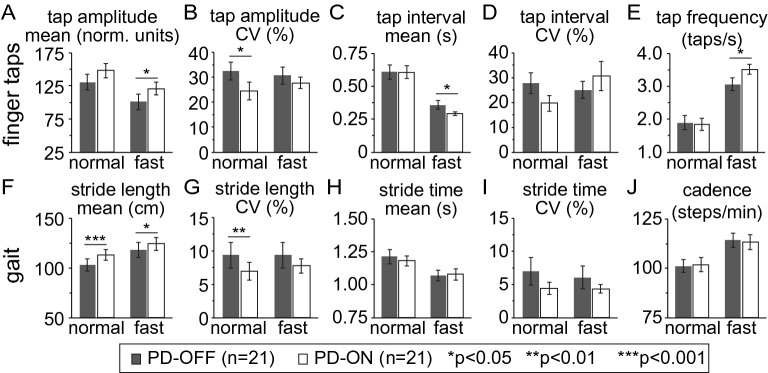
Levodopa responsiveness of spatiotemporal parameters of upper limb finger tapping and gait. Levodopa responsiveness in upper limb spatiotemporal parameters of mean and variability (CV) in finger tap amplitude (**A**, **B**), finger tap interval (**C**, **D**), and tap frequency (**E**) are shown in the top row for PD participants OFF levodopa (PD-OFF) and ON levodopa (PD-ON). Levodopa responsiveness in gait spatiotemporal parameters of mean and variability (CV) in stride length (**F**, **G**) stride time (**H**, **I**), and cadence (**J**), are shown in the bottom row. Bar graphs are plotted as the mean ± SEM.

Mean finger tapping amplitude (Fig. 4A) and gait stride length (Fig. 4F) significantly increased with levodopa at fast speed, and although they both also increased at normal speed, the increase in mean tap amplitude was not statistically significant (Fig. 4A, normal). Tap amplitude and stride length variability (CV) also decreased in the levodopa ON-state (Fig. 4B,G) at normal speed, but not statistically so at fast speed. Of our secondary and exploratory measures, mean tap interval was shorter ON vs OFF levodopa (Fig. 4C) at fast speed without corresponding change in mean stride time (Fig. 4H). There were no significant differences ON compared to OFF levodopa in tap interval or stride time variability (Fig. 4D,I) or tap frequency and gait cadence (Fig. 4E,J).

**Figure 5:**
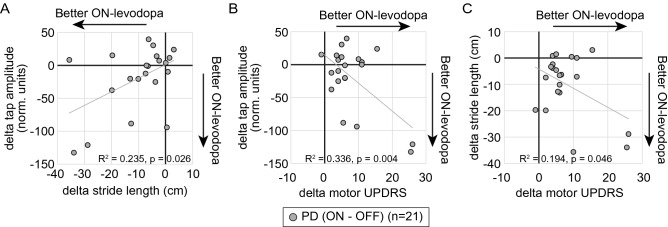
Comparison of levodopa response in tapping amplitude, gait and motor UPDRS scores in participants with Parkinson’s disease. Mean change in amplitude measures of finger tapping, gait stride length and motor UPDRS scores between the ON and OFF conditions are shown for (**A**) tap amplitude vs stride length, (**B**) tap amplitude vs motor UPDRS scores and (**C**) stride length and motor UPDRS scores. Each symbol represents a single participant. Best fit lines are also shown.

In order to compare the magnitude of levodopa response in both finger tap amplitude and stride length in each individual, and determine if they were also related, we calculated the difference between the OFF and ON result (delta) for finger tapping amplitude and gait stride length at normal speed. The delta tap amplitude was predicted by the delta stride length (F(1,20) = 5.827, R^2^ = 0.235, *p* = 0.026) and they were positively correlated (Pearson’s = 0.484) (Fig. 5A). Gender adjusted unstandardized residuals for delta tap amplitude and delta stride length were also significantly related (F(1,20) = 5.658, *p* = 0.028, R^2^ = 0.229; Pearson’s = 0.479).

As the motor UPDRS is used in the clinical setting to gauge motor improvement with levodopa, we also wanted to determine whether this overall motor improvement was related to the gait and finger tap amplitude measures. We therefore calculated each participants’ levodopa response on the motor UPDRS (delta). The delta motor UPDRS also predicted delta tap amplitude (Fig. 5B) (F(1,20) = 10.949, R^2^ = 0.366, *p* = 0.004), and delta stride length (Fig. 5C) (F(1,20) = 4.576, R^2^ = 0.194, *p* = 0.046) even after adjusting for gender and comparing unstandardized residuals. Since higher motor UPDRS scores correspond to worse disease, as expected there was an inverse correlation between delta motor UPDRS and both delta tap amplitude (Pearson’s = − 0.605) and delta stride length (Pearson’s = − 0.441). These were significant adjusting for the multiple comparisons.

## Discussion

Amplitude setting is impaired in PD, whether it be in the form of bradykinetic finger or foot tapping, micrographic handwriting, or decreased stride length when walking. One of the primary findings of our study was that amplitude setting of the more disease affected side during bimanual anti-phase tasks in the upper limb and lower limb were correlated and showed a similar response to motor load and to levodopa. This finding is important for multiple reasons. Firstly, PD patients are often divided into two groups clinically: (1) upper body or tremor predominant Parkinsonism, with tremor and/or upper limb bradykinesia being predominant features, or (2) lower body Parkinsonism with shuffling gait and postural instability. Since our study suggests that the severity of amplitude deficits are comparable in both upper and lower limbs in individuals, focusing on the predominant disease feature while planning treatments may limit overall functional improvement. Secondly, from a research standpoint, pathways for modulation of gait are difficult to study; functional magnetic resonance imaging (fMRI) cannot be performed while walking. However, our finding that the response to motor load and levodopa affects amplitude setting similarly both in finger tapping and gait, suggests that upper limb bimanual movements could be used as a simpler model system.

While gait control also involves balance control circuity not activated during finger tapping, our finding that smaller tap amplitudes and shorter stride length were correlated in the same PD participants, suggest that common circuits modulate amplitude during bilateral coordinated movement. Levodopa improved both tap amplitude and stride length, at least in the fast conditions, suggesting that dopaminergic pathways are involved. Stride length is shorter in PD^27^, and a sequential decrease in stride length (termed the sequence effect) has been suggested as a mechanism for freezing of gait episodes^25,28^. As in other studies^9,20^, we find that PD participants, at least in the MA hand, have smaller tap amplitudes. Whether a similar sequence effect leads to upper limb freezing episodes as it does in gait remains to be seen.

Besides absolute amplitude setting, variability or arrhythmicity in movement amplitude between successive repetitive movements is also important in PD. In our study we report an increase in tap amplitude variability in the MA (but not LA) hand in PD compared to control, similar to that seen using a MIDI keyboard tapping task^29^. While we did not find a correlation between the CV in finger tapping amplitude and gait stride length CV in PD-OFF, both improved in the levodopa ON-state (PD-ON), suggesting that upper limb and gait rhythmicity may be modulated through common dopaminergic pathways as well.

PD is an asymmetric disease, usually starting unilaterally with maintained asymmetry even with progression to bilateral disease. In our study, we were able to objectively measure the asymmetry in PD upper limb function when compared with control participants. Consistent with our findings, a prior study using a MIDI keyboard reported decreased tap velocity in the more affected hand in untreated PD participants within 1.5 years of diagnosis^10^. Dual-task behaviors have also been shown to preferentially affect performance in the more affected compared to less affected hand^30^. Asymmetry index calculations often utilize right and left limbs^31^ but not more relative to less affected limbs, so care must be taken when interpreting results.

Prior studies have not addressed whether levodopa affects upper limb movement similarly to gait. To address this deficiency, we performed upper limb and gait measurements in the same people on the same day. We found that levodopa increased finger tap amplitude and stride length, especially in the fast speed condition. Variability in finger tap amplitude and stride length were also reduced by levodopa. Importantly, the magnitude of these changes was correlated between the upper limb and gait measures. Prior studies of levodopa effects on upper limb repetitive movements have been mixed. Amplitude measures have been shown to improve^7^ or remain unchanged with levodopa^16,20^. Tap speed has been shown to improve^32^. Tap interval was decreased by levodopa in one study^32^ but unaffected in others^16,20^. Barbe and colleagues^16^ also show levodopa responsiveness in stride length in their participants but unlike in our study, they do not directly compare responses to upper limb measures.

The common pathways affecting both upper and lower limb amplitude setting are not clearly defined. However, cerebellar connectivity may be an important area to explore in future studies. For example, a recent exploratory meta-analysis suggested that the cerebellar locomotor region showed the most consistent gait-related activation in PD^33^. Two finger tapping studies, one using a sequential finger tapping task^34^, and another using a motor timing task also showed greater activation of the bilateral cerebellum^35^. Based on our results, an upper limb task-based paradigm using dynamic fMRI, while manipulating the amplitude of finger tapping might help define network level changes responsible for impaired amplitude setting common to gait and finger tapping. This could in turn help development of therapies to treat levodopa resistant bradykinesia/tachykinesia and gait deficits.

There were some limitations to this study. As a single size data glove was utilized, we cannot exclude sensor location increasing variability in finger tapping measures, but the glove fit snuggly on most and hand size is not affected by PD. Our groups did show different gender distributions but subgroup analysis and adjusting for sex differences did not affect our results. Due to the number of parameters tested, we may be overestimating the number of variables that showed statistical significance, despite attempts to account for these issues. Our study was not designed to compare our method of upper limb kinematic measurement to prior studies using different methods. However, analogue encoders placed on the rotation axis of the index finger have previously been used^36^, and the results showed decreased amplitude in finger tapping in PD compared to controls, levodopa responsiveness in finger tapping amplitude and correlation with UPDRS scores correctly reflect disease pathology.

## Conclusions

In summary, upper limb and gait amplitude setting in PD are scaled similarly with dopamine supplementation and motor demands. This suggests these automated motor functions are subserved by common functional networks.

## Supplementary Information

Supplementary Information 1.

## Data Availability

The datasets generated during and analyzed during the current study are available from the corresponding author on reasonable request.
